# Utilizing AI for extracting insights on post WHO's COVID-19 vaccination declaration from X (Twitter) social network

**DOI:** 10.3934/publichealth.2024018

**Published:** 2024-03-18

**Authors:** Ali S. Abed Al Sailawi, Mohammad Reza Kangavari

**Affiliations:** 1 School of Computer Engineering, Iran University of Science and Technology, Tehran, Iran; 2 College of Law, University of Misan, Amarah, Iraq

**Keywords:** AI, Twitter feeds, COVID-19, crisis response, WHO, vaccination, decision support system, public sentiment, real-time data analysis

## Abstract

This study explores the use of artificial intelligence (AI) to analyze information from X (previously Twitter) feeds related to COVID-19, specifically focusing on the time following the World Health Organization's (WHO) vaccination announcement. This aspect of the pandemic has not been studied by other researchers focusing on vaccination news. By utilizing advanced AI algorithms, the research aims to examine a wealth of data, sentiments, and trends to enhance crisis management strategies effectively. Our methods involved collecting a dataset of tweets from December 2020 to July 2021. By using specific keywords strategically, we gathered a substantial 15.5 million tweets, focusing on important hashtags like #vaccine and #coronavirus while filtering out irrelevant replies and retweets. The assessment of three different machine learning models–BiLSTM, FFNN, and CNN – highlights the exceptional performance of BiLSTM, achieving an impressive F1-score of 0.84 on the test set, with Precision and Recall metrics at 0.85 and 0.83, respectively. The study provides a detailed visualization of global sentiments on COVID-19 topics, with a main goal of extracting insights to manage public health crises effectively. Sentiment labels were predicted using various classification models and categorized as positive, negative, and neutral for each country after adjusting for population differences. An important finding from the analysis is the variation in sentiments across regions, for instance, with Eastern European countries showing positive views on post-vaccination economic recovery, while China and the United States express negative opinions on the same topic.

## 1. Introduction

The COVID-19 pandemic has presented unprecedented challenges worldwide, demanding swift and informed decision-making to effectively respond to the ever-changing situation. Extracting insights from diverse sources, including social media platforms like Twitter, has become essential in comprehending the virus's impact and making well-informed decisions. By harnessing advanced technologies such as artificial intelligence (AI) and data analytics, decision-makers can derive valuable insights from the vast amount of data available on social media platforms. One crucial area of focus is sentiment analysis, which involves analyzing the emotions and attitudes expressed in tweets and other online content related to COVID-19 [1]–[3]. Sentiment analysis offers valuable information on public opinion, concerns, and reactions towards the pandemic [3]. By examining the sentiment of tweets, decision-makers can gauge the level of anxiety, fear, or optimism among the population. This information can aid in tailoring communication strategies, addressing concerns, and providing timely and accurate information to the public [1]–[3]. In this study, we explored an efficient approach to sentiment analysis using a COVID-19 dataset obtained through the Twitter API during the WHO announcement of vaccines such as #Pfizer, #Moderna, and #AstraZeneca to combat the pandemic. Our proposed framework automates data labeling through LDA topic modeling and utilizes Bert embeddings for feature vector construction to classify the data contextually. The classification methods employed BiLSTM, CNN, and FFNN models, with BiLSTM showing the best performance in our comparisons. Training sets were used to teach the classifier text features, while test datasets evaluated classifier performance, with a 70% split for training and testing. Sentiment labels were predicted using various classification models, categorized as positive, negative, and neutral for each country after normalization by population. The sentiment analysis across countries was conducted using BiLSTM in our study.

Amid the COVID-19 pandemic, numerous research studies have focused on conducting sentiment analysis to effectively manage public health [4]. These analyses encompass mental health assessments [4], evaluations of economic impacts [4], public perceptions regarding COVID-19 restrictions, success stories from other countries in reducing illness rates, strategies to mitigate virus spread [5], and methods to alleviate anxiety surrounding the pandemic. These interconnected topics can elicit both negative and positive responses in different countries based on their unique cultures and government policies. Understanding public opinions during crises is crucial for decision-making, with social media serving as a valuable tool for gauging sentiments [6]. Platforms like Twitter provide insights into people's emotions regarding various pandemic-related issues such as job losses, family separations, income reductions, and other related impacts. Some segments of society have leveraged the pandemic to bolster economic conditions and enhance quality of life. Notably, certain athletes have publicly opposed vaccination, influencing community attitudes towards vaccination efforts. The popularity of these athletes can sway public willingness to receive vaccines, highlighting the importance of their presence in societal decision-making [7]. This research delves into Twitter data to explore community attitudes towards vaccination, assessing the impact of public vaccination announcements on living standards.

The global impact of COVID-19 on economic conditions, rising unemployment rates, business closures, mental health effects, and the influence of vaccine announcements on public opinion has not been thoroughly investigated. Therefore, there is a need to devise a comprehensive data analysis strategy to bridge this knowledge gap. This study presents a new framework to address these challenges, featuring an automated labeling approach and employing Bert embeddings with deep learning models for classification. This framework is specifically designed to handle short, user-centric, and colloquial text related to various crises. The primary objective of this framework is to minimize human labeling expenses and provide an automated approach for improved data categorization. Furthermore, the research introduces sentiment analysis for specific topics as an innovative method to better comprehend the economic implications of COVID-19. The article is structured as follows: Section 2 focuses on related works and literature review. In Section 3, the proposed method is introduced, outlining the topic extraction process and explaining concepts like datasets, LDA, tokenization, lemmatization, bigrams, trigrams, and artificial intelligent architects. Section 4 assesses the performance of the current work through result comparisons, *t*-test evaluation for hypothesis coherence, ROC and AUC score evaluations, and other criteria such as F1-score. Section 5 delves into detailed investigations of sentiment results for each country based on dominant topics. The research questions explore the effectiveness of AI algorithms in analyzing COVID-19 Twitter data for crisis management, compare machine learning models' performance on sentiment analysis, and investigate global sentiment variations to improve AI-driven decision support systems for public health crises.

## 2. Related works

Our study investigates the impact of the WHO's public vaccination announcement on public mental health during the global pandemic, taking into account economic challenges and government restrictions affecting societal well-being. We focus on real-time data collection following the WHO announcement, utilizing AI for efficient sentiment analysis. Our main goal is to comprehend the economic effects of COVID-19, create automated sentiment analysis tools, and offer crucial crisis management insights for decision-making in the post-vaccination phase, advancing AI-driven systems for public health emergencies. In our related research, we examined existing studies to identify gaps and trends. Initially, we conducted a literature review on time periods and data size in COVID-19 datasets. Subsequently, we analyzed articles that employed methodologies similar to ours, categorizing them based on index usage and language in COVID-19 datasets. Finally, we reviewed literature on vaccinations and AI approaches in COVID-19 datasets.

Xue J and colleagues [8] conducted an analysis of a dataset comprising 4 million COVID-19-related tweets from March 1 to April 21, 2020. They utilized the Latent Dirichlet Allocation (LDA) technique to identify significant themes within the COVID-19 dataset. These themes encompassed public health policies, social distancing, updates on COVID-19 cases and fatalities, the situation in the USA, and global COVID-19 cases. In a separate study, Xue J et al. [9] employed LDA to identify 10 distinct themes using a dataset of 1.9 million English-language tweets from January to March 7, 2020. These themes included updates on confirmed cases, COVID-19-related deaths, global cases outside of China, the outbreak in South Korea, the early stages of the illness in New York, public health concerns, newly recovered cases, economic challenges, preventive measures, governmental decisions, and public transmission chains. Furthermore, they conducted sentiment analysis, which revealed a prevailing sense of fear associated with the unknown nature of COVID-19 across all identified themes. Their work suggested that sentiment analysis could inform decision-making in public health policies, such as proposing new designs for masks and implementing social distancing in public spaces. While their work involved manual and time-intensive sentiment analysis, our current study enhances sentiment analysis by proposing automatic labeling for more efficient detection.

Hung M et al. [10] employed machine learning techniques to examine sentiment analysis expressed in Twitter data during the COVID-19 pandemic, focusing on the USA from March 20 to April 19, 2020. Using Latent Dirichlet Allocation (LDA) for topic modeling, they identified five primary issues within COVID-19 discussions, each associated with a spectrum of sentiments ranging from positive to negative. These discussions encompassed public health, emotional behaviors, trading during challenging circumstances, social alternatives, and stress-inducing situations such as relationship breakdowns. Their sentiment analysis of 902,138 English-language tweets revealed that 48.2% expressed a positive viewpoint, 31.1% held a negative opinion, and 20.7% did not express a clear opinion. Understanding public mental health regarding these topics could inform decision-making in mental health policies. Similarly, Imran A S et al. [11] utilized various deep learning approaches to analyze COVID-19 tweets from six highly populated countries: India, Norway, Pakistan, Sweden, USA, and Canada. They trained their dataset using BiLSTM, BERT, GloVe, and GRU methods to conduct sentiment analysis and emotion detection in these countries. Furthermore, Chandrasekaran R et al. [12] compiled a dataset comprising 13.9 million COVID-19-related tweets from January 1 to May 9. They identified 10 issues using LDA and evaluated the sentiment analysis and scoring of each topic. Their findings indicated that tweets discussing the increase in the number of cases, the origin of the COVID-19 outbreak, and political decisions regarding COVID-19 restrictions exhibited an overall negative sentiment. Conversely, a shift from negative to positive viewpoints was observed in tweets discussing prevention measures such as wearing face masks, the detrimental impact on trade, the strain on hospital staff, and post-illness treatment and recovery. Public health decision-making can be enhanced through the consideration of sentiment analysis. While the aforementioned studies focused on specific periods during the COVID-19 pandemic, they did not address the early stages of the WHO announcement of public vaccination. Our current work addresses this gap by applying a rapid and effective model to sentiment analysis.

Yin H et al. [13] compiled a dataset of 13 million COVID-19-related tweets collected over a two-week period to conduct sentiment analysis on extracted topics. Their findings indicated an overall more positive sentiment than negative opinion. Additionally, their topic-level sentiment analysis revealed that topics such as ‘stay_home’ and ‘stay_safe’ were associated with positive sentiment, while the ‘people death’ topic had a negative sentiment. This suggests that in public health policy, there is a focus on improving life standards and enhancing public health. On the other hand, Abd-Alrazaq A et al. [14] identified 12 topics categorized into 4 issues: the origin of virus spread, its manual or non-manual sources, its impact on public health, and trading, and decision-making to mitigate infection risk and spread. Their sentiment analysis on public health showed that 10 topics had a positive sentiment, while two topics, namely deaths caused by COVID-19 and increased racism, had a negative sentiment.

Numerous studies, including those referenced in this research, have conducted sentiment analysis related to public health in Arab countries. Most of these studies have involved limited analyses within a specific period, primarily focusing on Arabic tweets. For instance, Aljabri M et al. [15] examined the opinions of Twitter users in the Arab region of the Middle East. Their results focused on segregating the opinions of male and female populations, analyzing the sentiment within each country's population. We incorporated our findings in the results section, aligning with their approach. Alqurashi S et al. [16] compiled over 8000 COVID-19-related tweets from Arabic countries and categorized them manually, using various machine learning models to detect misinformation or fake news about the spread of the COVID-19 pandemic. In contrast, Ameur M S H and Aliane H [17] trained over 10,000 Arabic tweets related to COVID-19 into 10 distinct categories, such as instigating hate among couples and the public, lack of a cure, declining morals, fake news or opinions, unemployment and trade disruption, negative speech, and reduced income. In other words, their work focused on the negative aspects of the COVID-19 situation during the peak outbreak. Ameur M S H and Aliane H [18] also developed a dataset of 5000 tweets from Arabic countries, manually classifying them as either sarcastic or not. Similar to other researchers, they trained their work using various machine learning models. Alsudias L et al. [19] conducted a study where they utilized K-means clustering to identify various topics discussed on Twitter during the pandemic. These topics included COVID-19 situations, locations, limitations, and prevention measures. Additionally, the researchers manually sampled 2000 tweets to identify rumors and classified them as containing positive information, fake news, or being unrelated to the COVID-19 pandemic.

Another study examined the psychological impact of COVID-19 to assess human behavior trends [20]–[21]. It revealed that individuals are experiencing a crisis due to the coronavirus, leading to heightened anxiety levels fueled by COVID-19-related news. In a separate investigation, Polyzos E et al. [22] explored the effects of online campaigns honoring frontline workers on COVID-19 outcomes in the United Kingdom. By employing random regression forests and cointegration analysis on Twitter data, they identified delayed negative impacts from unfavorable sentiments to vaccination rates and from new cases to negative sentiment posts. Mittelmeier J and Cockayne H [23] focused on supporting international students during crises like COVID-19, utilizing Twitter data to combat discrimination and promote inclusive public backing. Their study highlighted the evolving perceptions of international students, shifting from being viewed as potential disease carriers to recipients of empathy and support following campus closures, underscoring the importance of addressing racism and negative portrayals in higher education. Chen J et al. [24] examined public sentiments towards border openings and international travel post-COVID-19, particularly analyzing the Australia-New Zealand travel bubble and identifying eight emotional responses expressed on social media. They observed positive reactions such as anticipation, joy, and trust, alongside increased fear levels during temporary travel bubble suspensions, offering valuable insights for tourism recovery and crisis management. Lastly, Carvache-Franco O et al. [25] investigated communication patterns during tourism crises amid the COVID-19 pandemic using Twitter data, emphasizing the effectiveness of Twitter in crisis communication for tourism businesses.

Regalado-Pezúa O et al. [26] studied the influence of social networks and traditional media on consumer behavior during the COVID-19 pandemic in Peru, observing shifts in purchasing behavior without direct correlation to negative emotions. Polyzos E [27] leveraged social media data for real-time decision-making during the war in Ukraine, noting varied market responses to conflict escalation, highlighting the value of user-generated content in critical decision-making processes during war events.

Mandal R et al. [28] utilized transformer-based sequence modeling to classify topics and analyze sentiment in short texts extracted from user tweets related to the Great Barrier Reef. Their research yielded promising results, offering valuable insights for researchers dealing with the classification of extensive datasets and numerous target classes. This approach of employing advanced techniques like text mining and transformer-based architectures on data from various channels, such as social media, proves beneficial in facilitating informed decision-making amidst data intricacies and uncertainties.

Yousefinaghani S et al. [29] investigated public sentiments towards COVID-19 vaccines using Twitter data, analyzing over 4.5 million tweets from January 2020 to January 2021 to compare sentiments over time, geographical distribution, themes, and engagement metrics. They found a prevalence of positive sentiments with higher engagement, noted more discussion on vaccine rejection than interest in vaccines, and identified Twitter bots and activists as sources of vaccine opposition. Hayawi K et al. [30] developed a machine learning framework for detecting COVID-19 vaccine misinformation, achieving a high F1-score using the BERT model. Bonifazi G et al. [31] proposed a multilayer network approach to study discussions on COVID-19 vaccines on Twitter, highlighting differences in network structures between anti-vaxxers and pro-vaxxers, emphasizing the importance of influential users in analyzing such discussions.

One of the significant contributions of our current study is the exploration of the impact of the WHO's announcement regarding public vaccinations on public mental health during the challenging global pandemic situation. These difficulties can be understood as stemming from economic hardships and their repercussions on families' well-being, or as government-imposed restrictions aimed at curbing the spread of the pandemic and their effects on society's mental health. Our key innovation in this study is the collection of data immediately following the WHO's announcement on public vaccinations during the COVID-19 pandemic, specifically focusing on sentiment analysis for public health. Moreover, our AI approach introduces a novel data labeling method to expedite insights extraction. Unlike many existing studies that rely on manual sentiment analysis requiring significant time investments, our work emphasizes real-time analysis, addressing the delay in sentiment analysis through our proposed methodologies. Another unique aspect of our research is the application of sentiment analysis during the early stages of the WHO's vaccination announcement period, offering potential support for informed decision-making by governments. Our approach transcends geographical boundaries, concentrating solely on language detection among Twitter users.

## 3. Proposed framework

**Figure 1.**
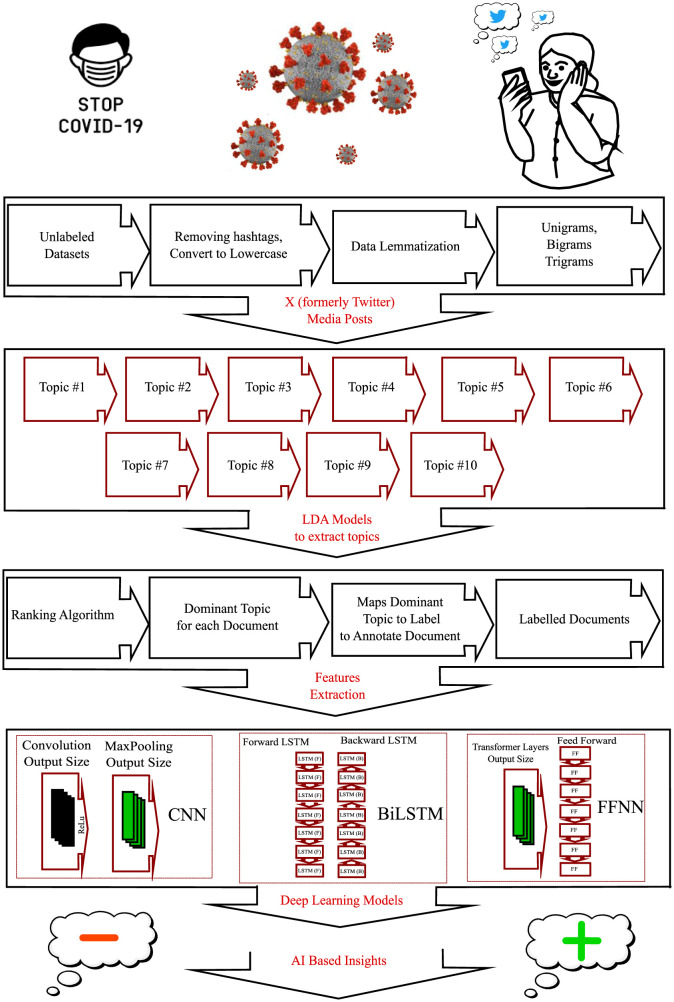
Proposed framework detail with each component.

To achieve accurate classification results, it is important to label datasets and use appropriate classification models with contextual embeddings. We start by using automated labeling through LDA topic distributions [32]–[34]. LDA topic refers to the topics discovered by the LDA algorithm in natural language processing, which identifies hidden topic structures based on word distribution. This technique helps us understand the main themes in a collection of documents without prior knowledge. These LDA topics are valuable for tasks like document clustering, topic modeling, and information retrieval. We then introduce a new ranking algorithm to prioritize these topics for dominant topic extraction. Next, we extract features using Bert embeddings, which capture the contextual meaning of words using the BERT model. These embeddings preserve contextual information and can be used as input features for various natural language processing tasks such as text classification, named entity recognition, and sentiment analysis. Figure 1 illustrates the framework we propose, which outlines the steps we will discuss in more detail in this section.

### 3.1. Datasets

The primary objective of this study is to analyze the viewpoints of social media users regarding the World Health Organization's (WHO) announcement about COVID-19 vaccination. On January 30, 2020, the WHO Director General declared the novel coronavirus outbreak as a Public Health Emergency of International Concern. By March 11, COVID-19 had been classified as a pandemic by the WHO. Since then, there has been remarkable progress in developing, producing, and distributing effective COVID-19 vaccines, some of which utilize mRNA technology. In December 2020, just one year after the first COVID-19 case was identified, the initial doses of COVID-19 vaccines were administered. The global rollout of COVID-19 vaccines has continued throughout 2021, with doses being distributed and administered across different continents. However, the efforts to control the pandemic face challenges due to disparities in vaccination coverage. As of July 2021, nearly 85% of vaccines have been administered in high- and upper-middle-income countries, with only 10 countries accounting for over 75% of the administered doses.

This research is centered on the analysis of tweets sourced from Twitter, a widely used social media platform boasting 353 million active users and over 500 million daily tweets. The dataset utilized in this study comprises tweets gathered from December 1, 2020, to July 31, 2021, accessed through the Twitter API. Pertinent tweets related to COVID-19 vaccines were acquired using specific keywords, limited to those composed in English. Replies, retweets, and quote tweets were omitted from the dataset. In total, 15,465,687 tweets were amassed, spanning various hashtags including #vaccine, #Pfizer, #Moderna, #AstraZeneca, #Sputnik, #Sinopharm, #coronavirus, #coronavirusoutbreak, #coronavirusPandemic, #covid19, #covid_19, #epitwitter, #ihavecorona, #StayHomeStaySafe, and #TestTraceIsolate. The dataset includes a variety of fields including tweet content, user account information, hashtags used, account location, tweet language, and retweet count. It is important to note that the dataset is specifically centered around English tweets (identified by the “Lang” field as “En”) and does not include retweets, although the number of retweets is factored into the analysis. The data showed good dispersion, indicating that our dataset covers a wide range of countries around the world.

**Figure 2.**
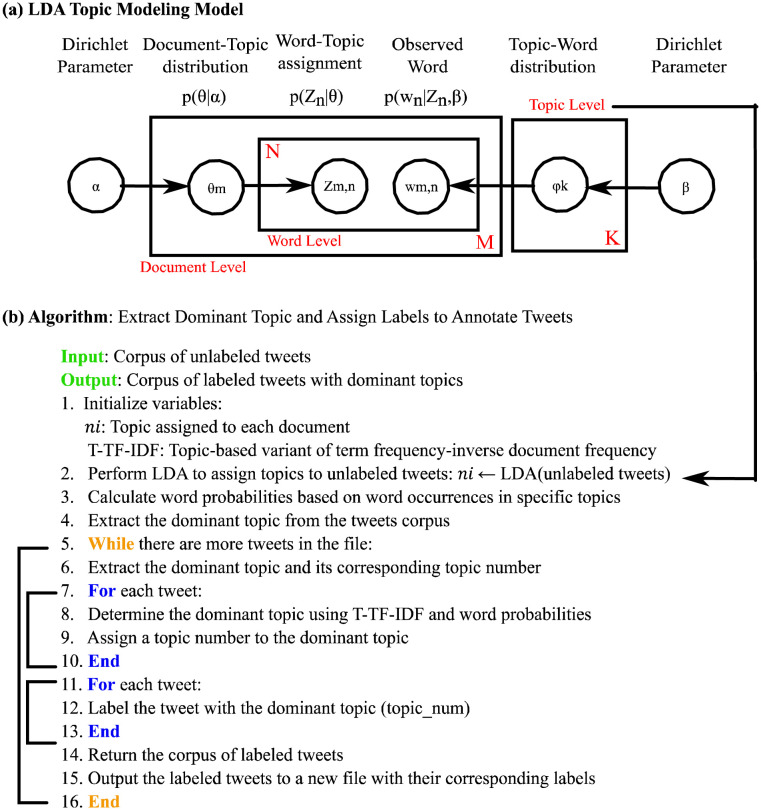
(a) LDA topic modeling model, (b) Extract dominant topic.

### 3.2. LDA model

In the Latent Dirichlet Allocation (LDA) model, topic proportions *θ*m are generated for each document in a collection using the alpha dirichlet distribution. The LDA model involves selecting topics and words based on these proportions. The *α* and *β* hyperparameters determine the prior distributions for the topic and word distributions, respectively. These hyperparameters shape the assignment of topics to words and documents in the LDA model [35]. In the realm of Latent Dirichlet Allocation (LDA), each document sample in set M is assigned topic proportions *θ*m from the alpha Dirichlet distribution, with random selection of topics *Z*_m,n_ and words *W*_m,n_ based on sample topic proportions and multinomial distributions *φ*_k_, respectively. The generalization process of LDA involves hyperparameters *α* and *β* determining Dirichlet priors on *θ*, where *θ*m represents topic distributions for all documents and *β* influences word distributions per topic. Variables M, N, and K indicate the total documents, words, and topics, with *φ*_k_ representing word distributions for topic K, *Z*_m,n_ indicating the word's topic in a document, *W*_m,n_ representing the specific word associated with a topic in a document, and *θ*m denoting the topic distribution of a document. The LDA Topic model have been showed in Figure 2 (a).

The algorithm presented in figure 2 (b) is followed to identify the main topic, which is then assigned as a label to annotate the tweets. Initially, the unlabeled tweets undergo preprocessing to prepare the dataset for further processing. Next, LDA is used to extract varying numbers of topics, and the topic coherence score helps determine the optimal number of topics. The topics are generated by LDA as a list of words with their respective probability scores within the topic. Each topic consists of different words with associated probability scores. Following this, the proposed T-TF-IDF ranking algorithm is utilized to identify the dominant topic from a list of topics for each tweet in the dataset. This process is repeated for all tweets in the dataset. After determining the dominant topic for each tweet, this topic serves as a label for annotation before being saved in a separate file. The resulting file contains tweets with labels represented by the dominant topics extracted from LDA and the ranking algorithm. The subsequent sections present comparisons of Tokenization, lemmatization, Bigrams, trigrams models with varying numbers of topics and their coherence scores.

### 3.3. Tokenization and lemmatization

Tokenization involves dividing a document or tweet into individual words or tokens. We used Spacy for tokenization and lemmatization, which provides detailed part-of-speech information and can handle sentence dependencies. After tokenization, we extracted words in their original forms using lemmatization. Unlike stemming, lemmatization considers the dictionary of the specific language, ensuring valid root forms. We focused on nouns, adjectives, and verbs during lemmatization, which can be beneficial for precise topic modeling using Latent Dirichlet Allocation (LDA).

### 3.4. Bigrams and trigrams

In this part of the article, we used Tokenization to divide documents or tweets into individual words or tokens, utilizing Spacy for tokenization and lemmatization. We also explored the use of bigrams and trigrams to maintain the semantic context in large texts, and how Gensim's phrases class was used to categorize phrases for the LDA method. Additionally, we discussed the extraction and labeling of topics using the LDA model, determining the optimal number of topics through measures such as coherence and prevalence scores. The LDA model was applied using various types of corpora, including unigrams, bigrams, and trigrams, in conjunction with a dictionary. The process of selecting the lowest and suitable number of topics for an LDA approach can be challenging and time-consuming. Two commonly used metrics for evaluating LDA models are full scores and rough scores. Coherence scores (full score) measure the semantic similarity between words in topics. They assess how well the words within a topic support each other and can be interpreted within a given context. Coherence scores are preferred for assessing LDA model performance because they provide human-interpretable topics. Perplexity scores, on the other hand, measure the model's prediction accuracy. A lower perplexity score indicates better prediction accuracy. However, recent studies have shown that perplexity scores may not always yield human-interpretable topics. Therefore, coherence scores are generally preferred for determining the optimal number of topics. To calculate coherence scores, the C_V coherence measure is commonly used. This measure considers a sliding window, one-set segmentation of top words, and measures like NPMI (Normalized Pointwise Mutual Information) and cosine similarity. The coherence score is calculated by summing the scores of word pairs (wi, wj) where i < j, and these words have the highest probability of occurring in the topic. The number of words considered for the overall score can be specified. In the evaluation of different topic numbers and variants of bigrams and trigrams on COVID-19, it was found that using bigrams with fewer topics yielded better and more coherent results. This is likely because social media data, which is often short, noisy, and sparse, tends to perform better with fewer topics. Coherence performance was also found to be superior with bigrams compared to trigrams. Further analysis revealed that topics with fewer words had higher coherence scores. This suggests that having a concise selection of words in each topic increases the likelihood of belonging to a specific topic. This is particularly relevant for social media text, which is typically limited to a maximum of 280 characters on platforms like Twitter. Upon examining topics, it was found that issues with high full relations values and frequently used words provided similar information. For example, in the COVID-19 Twitter dataset with bigrams and 10 topics, topic 4 had the highest coherence score and included important words related to lockdown, vaccination, masks, quarantine, and health. Once the optimal number of topics is determined, the next step is to extract a dominant topic from the LDA model topics to efficiently label each tweet automatically. The extracted topics from the COVID-19 Twitter dataset encompass information such as announcements, government and public health measures, and discussions on deficiencies in medical equipment in hospitals and hospital situations like HVAC systems. In conclusion, selecting the optimal number of topics for an LDA model involves evaluating coherence scores and considering the specific characteristics of the dataset. Fewer topics with a concise selection of words tend to perform better, especially for social media datasets. These extracted topics can then be used to label tweets and provide valuable information about different aspects of the dataset [36]–[38].

**Table 1.** Coherence measure values.

**LDA models**	**Coherence**
5 topics with bigrams	0.861
15 topics with bigrams	0.801
30 topics with bigrams	0.796
5 topics with trigrams	0.854
15 topics with trigrams	0.799
30 topics with trigrams	0.764

**Table 2.** Keywords and descriptions of extracted issues from our COVID-19 dataset.

**Topics/classes**	**Keywords**	**Description (Post-COVID-19)**
Topic 1	Disinformation, narratives, conspiracy, cover-up, new world, order, origins, Media	Conspiracy Theory
Topic 2	Updates, Reliable sources, Misinformation, Fact-checking	COVID-19 information
Topic 3	numbers, Death, Recovery, Vaccination, Variants, Testing	COVID-19 statistics
Topic 4	Economic, recovery, unemployment, growth post-COVID-19, economic, revival, Inflation, economy, Consumer, inequality	Economics
Topic 5	Aware, false, misleading, hydroxychloroquine, Miracle, silver, essential, oils, high-dose, vitamin C, garlic	Fake Treatment
Topic 6	Lockdown, Quarantine, Social distancing, Stay-at-home orders, Travel restrictions, Curfew, Mask mandates, Testing, Contact tracing, Vaccination campaigns, public health guidelines, Economic stimulus packages, Business closures, Remote work policies, School closures	Governmental Measures
Topic 7	Analyzing, responses, Examining, health, vaccination, global, polarization, recovery, Governance, crisis	Politics
Topic 8	Protocols, Contact, Testing, strategies, isolation, Travel, campaigns	Public health measures
Topic 9	Leadership, Policy, Partisanship, Accountability, International cooperation	Stocking Up
Topic 10	development, Efficacy, safety, distribution, immunity, hesitancy, Long-term effects	Vaccine/Cure

### 3.5. Dominant topic

The LDA model is utilized to extract topics from documents and determine the dominant topic for each document using the TF-IDF technique. This aids in labeling unlabeled tweets by identifying their primary topic. The proposed algorithm involves pre-processing the tweet dataset, extracting topics with LDA, representing topics as word sets with associated probabilities, ranking topics with T-TF-IDF, and labeling and saving the tweets based on dominant topics. Additionally, the algorithm compares the coherence scores of different LDA models with varying numbers of topics. All models were trained using Python 3 on a system equipped with an Intel Core i7 11th generation processor, 16GB RAM, and Nvidia Tesla K80 GPU.

### 3.6. CNN model

**Figure 3.**
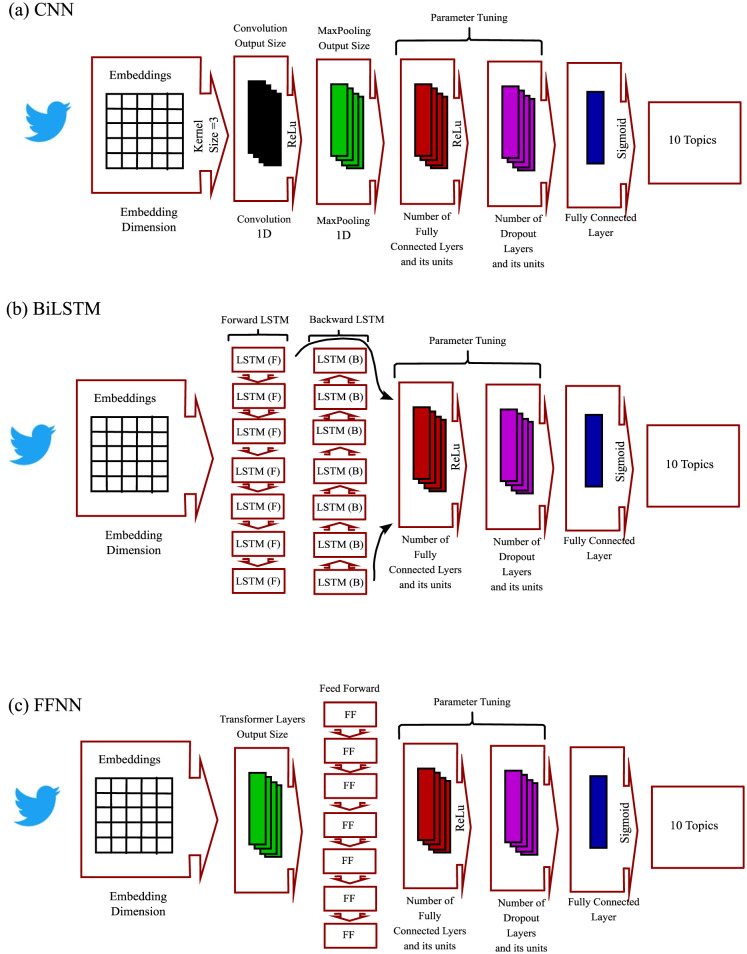
Present work deep learning models.

The CNN approach, illustrated in Figure 3 (a), has demonstrated strong performance in a range of NLP tasks. It starts with a word embedding layer using pre-trained FastText word embeddings to effectively represent tweets, including out-of-vocabulary sentiments. After the embedding layer, there is a 1D convolution section with an input size of n × 300, where n represents the number of words in the tweet and 300 is the dimension of the word embedding vector. Following this, a 1D maximum pooling section is used to reduce the features obtained from the convolution section. The model includes fully connected and dropout sections to prevent overfitting. In the final layer, 11 Sigmoid units are used for English, as tweets can belong to multiple classes. The model employs binary cross-entropy error as the loss function, suitable for multilabel classification, and applies the Relu activation function to all hidden layers.

### 3.7. BiLSTM model

The BiLSTM model, depicted in Figure 3 (b), is a type of recurrent neural network (RNN) known as Bidirectional Long Short-Term Memory (BiLSTM). It addresses the issue of vanishing gradients in RNNs. The architecture of the BiLSTM model resembles that of the CNN model, commencing with a word embedding layer that utilizes pre-trained FastText word embeddings to represent tweets. Following the embedding section, a bidirectional LSTM section is utilized, along with fully connected layers and dropout layers for regularization. The output layer of the BiLSTM model consists of a fully connected layer, with 11 Sigmoid units for English. Relu activation function is applied to all hidden layers, and the model uses binary cross-entropy error as the loss function, similar to the CNN model.

### 3.8. FFNN model

In Figure 3 (c), we can see our third model, the FFNN model. This model is known for its simplicity and versatility, making it effective for solving mapping problems. It consists of interconnected layers of neurons, with information flowing in a forward direction. However, it is not well-suited for handling sequential data. On the other hand, the BiLSTM model, a variant of the LSTM model, is capable of capturing both past and future information by processing the input sequence bidirectionally. This makes it suitable for tasks that require understanding the context and dependencies in sequential data. The CNN model, primarily used in image and signal processing tasks, can also be applied to sequential data analysis. It treats text as a one-dimensional signal and utilizes convolutional filters to capture local patterns and features. This makes it highly effective for tasks that involve understanding local relationships within a sequence. Both the BiLSTM and CNN models excel at capturing sequential dependencies, but the choice between them depends on the specific task and characteristics of the data. BiLSTM is more suitable for understanding long-range dependencies, while CNN is better at capturing local patterns and features. Relu activation function is applied to all hidden layers, and the loss function used is binary cross-entropy error, which is also employed in the CNN and BiLSTM models.

## 4. Performance evaluation measures

To validate our proposed framework, we conducted a classification task on our post-COVID-19 datasets. The goal was to assign tweets to one of 10 different classes, and we measured the performance using metrics like Accuracy, Precision, Recall, and F-measure. To analyze the results, we used a 10 × 10 confusion matrix, which visually represents the relationship between the predicted and actual labels for each tweet in the classifier. It's important to note that the values in the confusion matrix are calculated separately for each class. The matrix consists of True Positive (TP), False Positive (FP), True Negative (TN), and False Negative (FN) components. TP represents the instances where the classifier correctly predicted the correct class, while FP represents instances where false positives occurred. TN is the sum of values from all columns and rows excluding the evaluated class, and FN is the sum of values from the corresponding rows excluding TP for a specific class. We assessed the performance of the classifier using metrics like accuracy, precision, recall, and f-measure, which provide insights into its performance. The equations for calculating these metrics can be found in Table 3. Additionally, we calculated the true positive rate (TPR), also known as sensitivity, which is the probability that an actual positive will test positive, and the true negative rate, also known as specificity, which is the probability that an actual negative will test negative.

**Table 3.** Performance evaluation measures.

**Criteria**	**Equation**
*Precision*	$\frac{TP}{TP+FP}$
*Recall*	$\frac{TP}{TP+FN}$
*F1-score*	$2\times \frac{Precision\times Recall}{Precision+Recall}+measure$
*Accuracy*	$\frac{TP+TN}{TP+TN+FP+FN}$
*FPR*	$1-\frac{FP}{TN+FP}$

The F1-measure is a comprehensive metric that combines precision and recall to assess classifier performance. It ranges from 0 to 1, with 1 indicating perfect precision and recall. In addition to other evaluation metrics, classification tasks commonly utilize two curves: the ROC curve and the PR curve. The ROC curve plots the true positive rate (TPR) against the false positive rate (FPR), with TPR representing the recall measure or the proportion of correctly classified positive instances. FPR measures the proportion of incorrectly classified negative instances. On the other hand, the PR curve plots precision against recall, where precision is the ratio of true positives to the sum of true positives and false positives, and recall is the ratio of true positives to the sum of true positives and false negatives. These curves provide insights into the balance between true positive and false positive rates, as well as the trade-off between precision and recall. They assist in determining the optimal classification threshold and overall assessment of classifier performance. Additionally, the PR curve is a Precision-Recall curve that plots precision on the y-axis and recall on the x-axis.

The foremost limitation of prevailing text classification methodologies predominantly lies in their dependence on datasets annotated by humans, with only a handful incorporating datasets labeled automatically. Furthermore, these approaches exclusively rely on internal feature extraction schemes employed by deep learning classifiers. To ascertain the effectiveness of our proposed model, expounded in this scholarly paper, we undertake a comparative evaluation against several benchmark models, as delineated in Table 4. Another noteworthy achievement of this investigation is the graphical representation showcasing the dispersion of normalized sentiment scores across different countries for each categorized subject matter.

**Table 4.** A comparison of our proposed framework with baseline approaches utilized in previous studies on COVID-19 dataset.

**Authors**	**Approaches**	**Precision**	**Recall**	**F1-score**
Karami A et al. [39]	LIWC lexicon	71.8%	71.7%	70.2%
Gupta I et al. [40]	Clustering approach	69.1%	74.3%	75.3%
Abdul-Mageed M et al. [41]	Large COVID-19 tweets dataset, analysis, and classification	*	*	0.98%
Basiri M E et al. [42]	NBSVM model	0.858%	*	0.858%
Tripathi M [43]	nb svm and lstm	0.80%	0.79%	0.790%
present work	BiLSTM	0.85%	0.83%	0.84%

Following the comparative analysis, we introduced an innovative approach in our framework by implementing an automatic annotation method. For classification purposes, we employed transformer-based Bert embeddings in conjunction with deep learning-based classifiers such as CNN, FFNN, and BiLSTM. The evaluation results, presented in Table 4, demonstrate the superior performance of our model compared to baseline approaches on our disaster dataset. Specifically, the BiLSTM and CNN models showcased exceptional performance on the dataset, validating the efficacy of our proposed model in this study.

We performed a 10 × 3 cv paired *t*-test on our dataset to evaluate the statistical significance and validity of our proposed model classifiers compared to baseline models. This test is suitable for supervised classification learning when the same data is used for both the proposed and baseline approaches. For both methods, we utilized distinct COVID-19 datasets. The dataset was split into training and testing sets in a 10 × 3 cv paired t-test, with this procedure being repeated five times. Each iteration included a 30% split for training and a 70% split for testing. The process is then repeated with rotated test and train sets, resulting in two measures of performance difference. The 10 × 3 cv paired t-test is a statistical test that compares the performance of three classifiers. If the t-statistic value is greater than a predetermined limit, we can reject the null hypothesis and conclude that the classifiers' performance is significantly different. The MLxtend package provides an implementation of this test, calculating the t-values and p-values for the compared classifiers. The parameters of MLxtend require the names of the classifiers and the scoring mode, which, in this case, is mean accuracy. The statistical analysis indicates that all *p*-values in the dataset are less than 0.05, leading to the rejection of the null hypothesis. Furthermore, the baselines' values are notably lower than the threshold *p*-values. By examining the *t*-values, which surpass the limit value of 2.571, it is evident that the performance of the baseline methods differs significantly from the proposed framework. This leads to rejecting the null hypothesis that both models have similar performance. The statistical tests suggest that the model's performance is significantly superior to the state-of-the-art approaches that were compared.

## 5. Results

### 5.1. dataset results analysis and discussion

**Figure 4.**
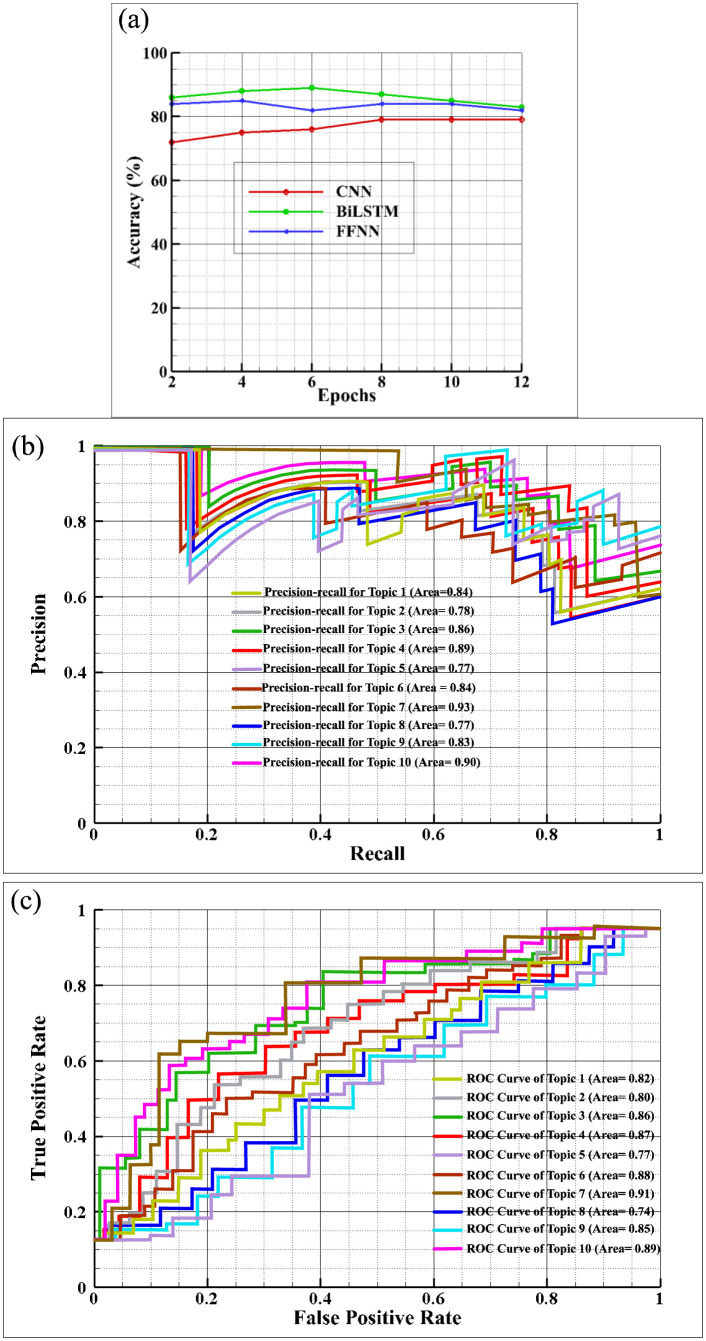
Performance metrics and graphs for classifiers on the dataset.

**Figure 5.**
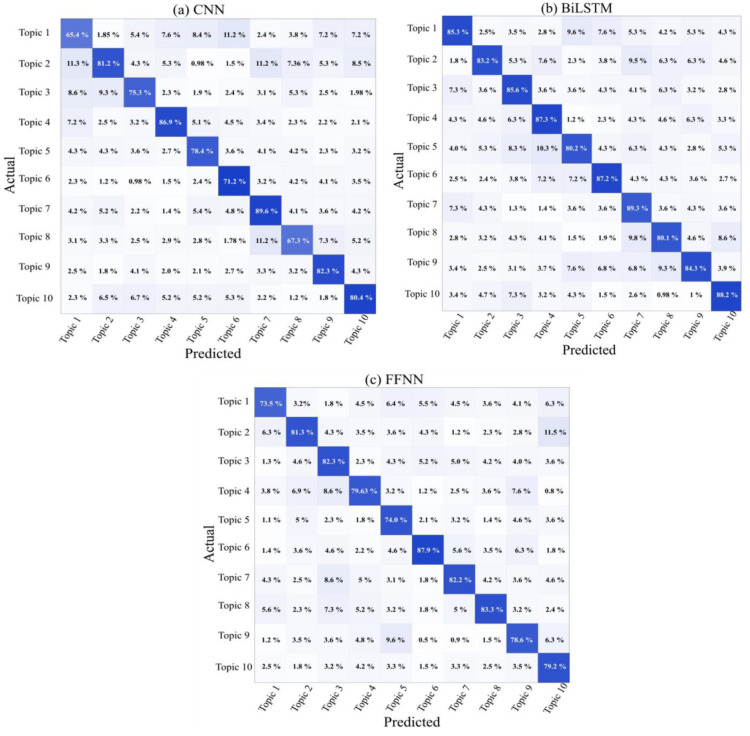
Confusion Matrix Values for COVID-19 Dataset across 10 Classes using Various Classifiers.

The proposed framework was applied to datasets collected after the COVID-19 pandemic, and the accuracy results are presented in Figure 4 (a). This figure illustrates the accuracy rates of various classifiers on the Post-COVID-19 dataset, as the number of epochs increases. Among these classifiers, the BiLSTM classifier achieved the highest accuracy of 89.02% after 6 epochs. The confusion matrix values in Figure 5 (b) show that the classification score for class 7 (Topic 7) is higher than the scores for other classes when using the BiLSTM classifier. Using the CNN classifier on the dataset, we obtained the highest accuracy score of 79.1% after 8 epochs. The analysis of accuracy rates in Figure 4 (a) shows a gradual decrease in the accuracy of the CNN classifier after 8 epochs. Meanwhile, the FFNN classifier achieved its highest accuracy of 85.15% after 4 epochs, but this accuracy declined in subsequent epochs, as depicted in Figure 4 (a). The confusion matrix in Figure 5 (b) illustrates the classification results for all 10 classes using the CNN classifier, with class 7 achieving the highest classification accuracy of 89.6%. While CNN was originally designed for image classification, it has been adapted for text classification, and the achieved accuracy level is satisfactory, as indicated by the analysis. Figure 5 (c) displays the confusion matrix values for all 10 classes when using the FFNN classifier on the dataset, revealing the highest classification rate of 87.9% for accurately classifying text into class 6 categories, closely followed by a class 8 accuracy rate of 83.30%.

Table 5 displays the precision, recall, and F1 measure scores for all classifiers. The comparison results indicate that the BiLSTM classifier outperforms the CNN and FFNN classifiers. This is because the dataset used in this study contains diverse data related to different situations of COVID-19, including slang words used by users in various contexts such as vaccination and life hope. Despite this variation in data, all classifiers exhibit higher precision scores compared to recall scores. Upon analyzing the classifiers individually, both BiLSTM and FFNN achieve higher scores than CNN. It is worth noting that even though FFNN has the advantage of maintaining hierarchical representation of text for longer durations, its scores are slightly lower (1% lower in F1) than those of BiLSTM. This suggests that the ability of BiLSTM to capture long-distance dependencies in texts is not as crucial in the COVID-19 dataset, which encompasses diverse data types that change over time. We evaluated the distinguishability between classes by creating ROC curves. Another way to evaluate the performance of a classification model is through the Precision-Recall (PR) curve. Precision measures the accuracy of positive class predictions, while recall measures the proportion of true positive instances classified correctly out of all actual positive instances. The PR curve demonstrates the tradeoff between precision and recall.

**Table 5.** Precision, recall, and F1 measure scores for our proposed model with each classifier on post-COVID-19 dataset.

**Classifiers**	**Precision**	**Recall**	**F1-score**
CNN	0.79	0.78	0.77
BiLSTM	0.85	0.83	0.84
FFNN	0.80	0.80	0.79

In Figure 4 (b), the PR curve is shown for all classes in our dataset using the highest accuracy classifier, which is the BiLSTM. The findings reveal a consistently high overall micro average area under the curve, indicating strong precision in effectively classifying the classes. Notably, Class 7 demonstrates superior precision and recall areas, signifying the classifier's adeptness in making accurate predictions with minimal errors for this specific class. Furthermore, Topic 10 and Topic 4 exhibit notable area scores of 0.90 and 0.89, respectively, showcasing the classifier's proficiency in accurately predicting these classes in numerous instances. Despite relatively lower scores for Class 5 and Class 8 compared to others, the precision curve remains elevated at specific thresholds, indicating the classifier's proficiency in accurately classifying instances for these classes as well. The ROC curve depicted in Figure 4 (c) for the COVID-19 dataset, utilizing the highest accuracy classifier, the BiLSTM, illustrates that Class 7 attains the highest AUC score of 0.91, surpassing other classes. This suggests the classifier's optimal classification of text into Class 7. Our analysis of the overall micro average and macro average indicates the exceptional ability of our classifier model in distinguishing between classes. The AUC areas for Topic 6 and Topic 10 are notably similar, with scores of 0.88 and 0.89, respectively, as evidenced by their closely aligned curves. The absence of overlap in these curves suggests their effectiveness in classifying categories.

### 5.2. Distribution of the analyzed tweets per country for each topic

**Figure 6.**
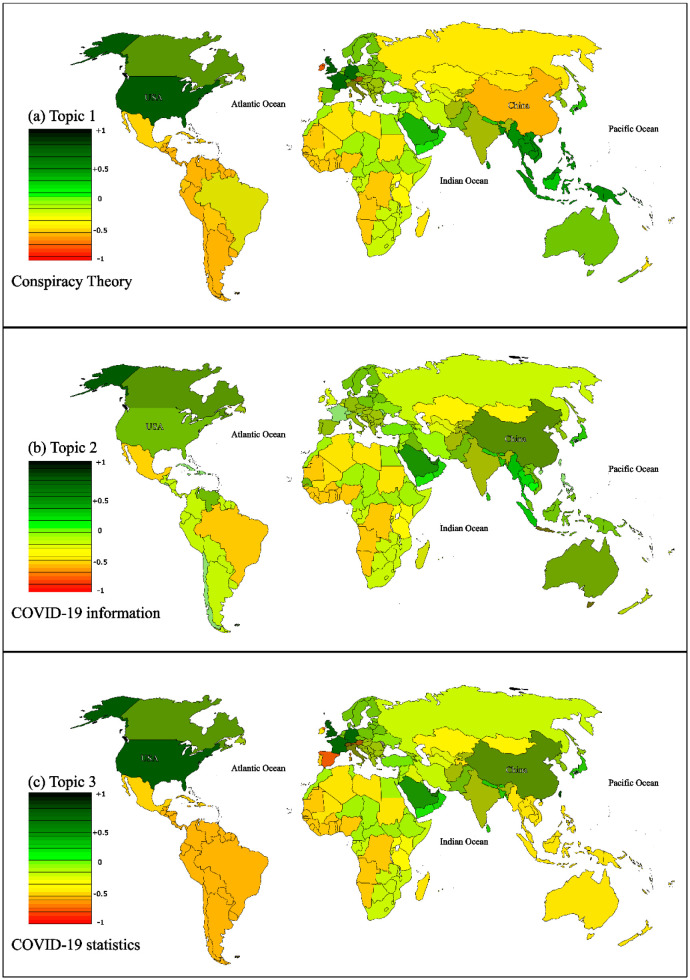
Normalized sentiment scores per country for topics 1, 2, and 3.

Upon conducting an assessment and identifying the topics, this section of the results focuses on the statistical analysis of each topic. Our primary approach, the BiLSTM model, was utilized to extract the distributions. As previously mentioned, tweets were categorized into 10 identified topics. The distribution of tweets analyzed per topic is visually represented in the accompanying figure. Notably, our BiLSTM model classified a significant portion of our data—32% of all 10 topics—as pertaining to Governmental Measures, while topics such as Stocking Up, Conspiracy Theory, and Fake Treatment [44] accounted for a minimal percentage, approximately 2%. This suggests that during the WHO announcement of public vaccinations, public opinion was largely centered around governmental limitations. The substantial percentage of tweets related to Governmental Measures indicates that individuals predominantly discussed personal matters concerning the pandemic and efforts to alleviate COVID-19 restrictions. Other topics, including COVID-19 News/Information, Public Health Measures, Vaccines/Cure, Politics, and Economics, accounted for 7%–20% of the tweets. Our analysis led us to the conclusion that many individuals believe that the reduction of governmental limitations would facilitate a return to work and enhance trade and income. Figure 6 illustrates the distribution of tweets per country for each topic, relative to the population of each country. The global community contributed the most to tweets about all 10 topics, underscoring the accuracy of our findings. When examining figures for each topic, we will consider the names of continents and regions. Our results indicated that in the Governmental Measures topic, most individuals held neutral or nearly negative opinions, signifying uncertainty about finding a solution regarding vaccination.

Figure 6 presents the distribution of normalized sentiment scores per country for three topics: Topic 1, Topic 2, and Topic 3. In general, all countries in North America displayed a positive sentiment towards Topic 1, Topic 2, and Topic 3. However, there were some countries that expressed a negative sentiment towards the categorized topic, as indicated in the figure 6. It is worth noting that the sentiment scores are normalized, meaning they have been adjusted to account for variations in sentiment measurement across different countries. This allows for a more accurate comparison of sentiment across regions. The sentiment analysis provides valuable insights into the global perception of these topics and can help inform decision-making and communication strategies. During the COVID-19 pandemic, the United States has embraced scientific evidence and official information over conspiracy theories, fostering trust and transparency with accurate data. Conversely, China has been more skeptical of COVID-19 conspiracy theories, emphasizing reliance on official information for public health management. Following the COVID-19 vaccination announcement, many Middle Eastern countries have shown a positive shift in their views on various topics. Despite facing significant challenges during the pandemic, Iran experienced higher rates of illness and job losses. However, after the WHO's COVID-19 vaccination announcement, Iran's perspective on these topics improved compared to other Middle Eastern countries.

The conversation around COVID-19 statistics offers valuable insights into public health, covering aspects such as post-vaccination new case data, mortality rates in the months following vaccination, recovery rates, types of vaccines, emerging variants, and increased COVID-19 testing. The issue of vaccinating children during the early stages of the pandemic was significant, with some parents expressing concerns about potential allergic reactions [45]. Figure 6 (c) demonstrates that in developed nations like the USA, COVID-19 statistics paint a positive picture of vaccination. However, more than 75% of African countries had low vaccination rates, highlighting disparities in global vaccination efforts. It's important to note that the impact of the pandemic differs between the USA and African countries. The early availability of vaccines in high GDP countries may have influenced negative attitudes toward COVID-19 statistics in African nations. This emphasizes the need for global vaccine access to achieve widespread immunity. The evolving information provided by the WHO during vaccination efforts is critical for public health, particularly from a mental health standpoint [46],[47]. Furthermore, the emergence of new COVID-19 variants has had minimal psychological effects on the public, likely due to the availability of pertinent vaccine information. Ensuring fair vaccine distribution globally and making informed decisions about vaccination are crucial for addressing cultural and social factors that impact vaccine management.

**Figure 7.**
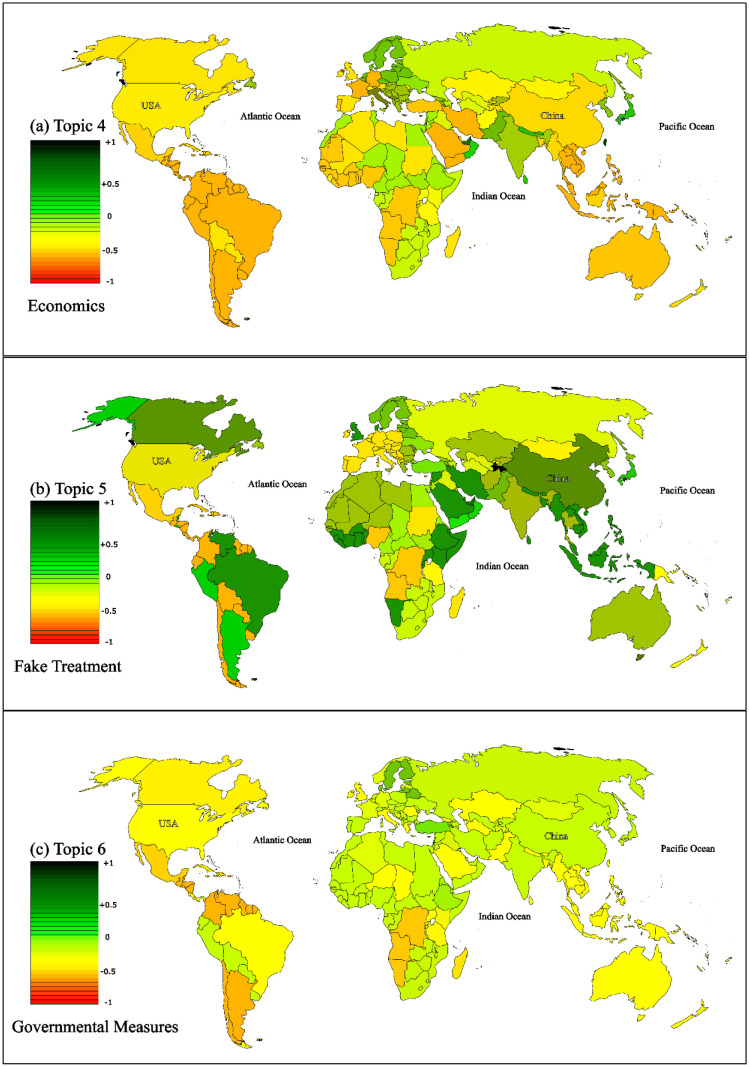
Normalized sentiment scores per country for topics 4, 5, and 6.

**Figure 8.**
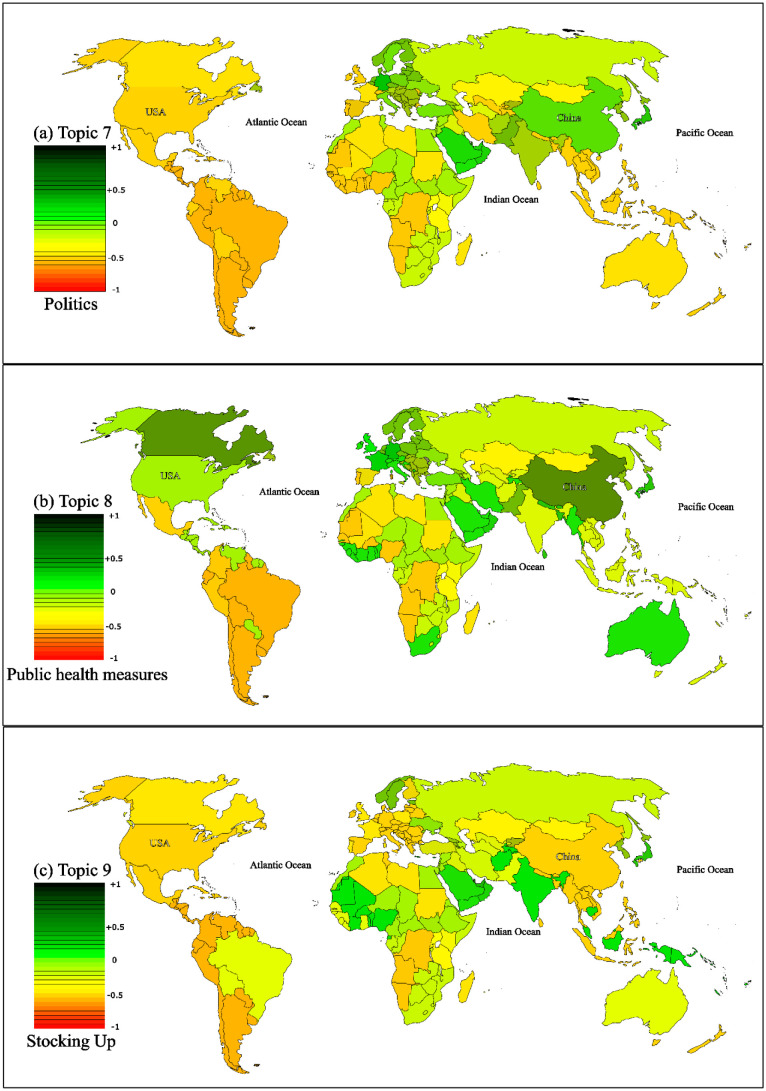
Normalized sentiment scores per country for topics 7, 8, and 9.

**Figure 9.**
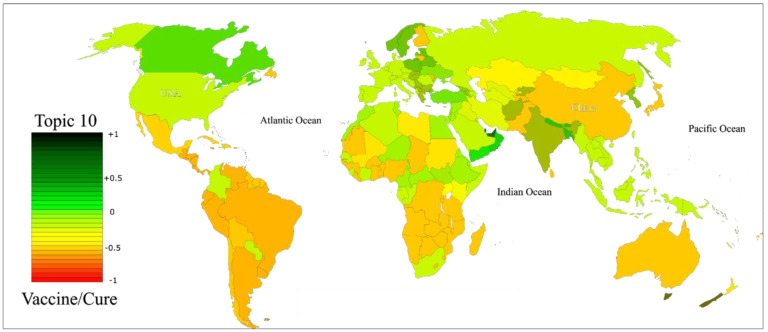
Normalized sentiment scores per country for topic 10.

Figure 7 displays the sentiment distribution per country for three topics: Topic 4, Topic 5, and Topic 6. The sentiment of users from the USA towards these topics was predominantly negative. However, other countries exhibited a more positive sentiment, as shown in the figure 7. It is worth noting that these sentiment scores reflect the growing optimism following the WHO's announcement on COVID-19 vaccinations. To validate our findings, we compared them with similar studies conducted in various regions, focusing on the emergence of the pandemic and its impact on user sentiment. Additional results for Topic 7 to 10 can be found in Figure 8 and 9, which depict the sentiment of users towards post-COVID-19 situations.

Topic 4 delves into economic sentiment analysis across various countries, with a specific focus on the United States and China. The analysis depicted in Figure 7 (a) indicates that China holds a more pessimistic view than the U.S. regarding economic recovery, unemployment reduction, income growth post-COVID-19, and inflation control. Walmsley T et al. [48] conducted a comparative study between the two nations, considering factors such as mandatory closures, gradual reopening processes leading to workforce declines due to health concerns, increased healthcare demand, reduced public transportation and leisure activity demand, potential telework resilience boosting communication services demand, and pent-up economic demand. Their research revealed that the United States is expected to incur GDP losses ranging from $3.2 trillion to $4.8 trillion over a two-year period due to COVID-19, surpassing China and other regions in terms of percentage decline. The primary driver behind these projections is the combination of Mandatory Closures and Partial Reopenings of businesses, which could significantly reduce U.S. GDP across various scenarios.

Furthermore, the study highlighted that Eastern European countries exhibit a positive attitude towards COVID-19 vaccinations, leading to increased trust in specific vaccine certifications. China's stringent measures, such as not opening airports to incoming travelers, aimed at maintaining zero COVID-19 cases, have resulted in trading restrictions for the country. In addition, following the reopening of several states in May, a considerable number of workers reentered the job market in sectors like restaurants, healthcare, and construction. This resurgence led to the creation of approximately 2.5 million jobs in May and 4.8 million in June, with fiscal policy support for small businesses playing a significant role in this job growth [49]. Consequently, the unemployment rate dropped to 13.3% in May and further to 11.1% in June. In June, unemployment rates among various demographic groups were 16.1% for Hispanics, 14.5% for African Americans, 13.8% for Asians, and 10.1% for whites. Notably, the unemployment rate remained higher for women compared to men during this period [49].

## 6. Conclusion

In this study, we leveraged advanced AI-based sentiment analysis to gauge public sentiment on Twitter following the COVID-19 vaccine announcement. The exceptional accuracy of our model in understanding sentiments underscores the potential of AI in extracting valuable insights from social media, especially during the COVID-19 pandemic and the subsequent vaccination phase. By employing sophisticated AI algorithms and machine learning techniques such as BiLSTM, we conducted a comprehensive analysis of sentiments and opinions on Twitter, providing valuable insights into public sentiment and regional considerations.

Moreover, the development of a fully automated Decision Support System (DSS) accessible through the Windows platform demonstrates the practical application of AI technologies in crisis management and decision-making. The high precision and efficient analysis time of our study underscore the effectiveness of the proposed framework. Overall, the findings of this research have the potential to significantly contribute to the development of AI-powered decision support systems for effectively responding to public health crises and emergencies, ultimately aiding in better-informed decision-making and crisis response.

Additionally, our research encompasses 10 distinct topics related to COVID-19 and its impacts, covering a wide range of aspects including disinformation, economic facets, fake treatments, governmental measures, public health, leadership, policy, and vaccine-related considerations. Notably, among the selected topics, class 7 demonstrated the highest AUC score of 0.91 compared to the other classes, with precision, recall, and F1-score criteria of 0.85%, 0.83%, and 0.84%, respectively. This indicates the robustness and accuracy of our analysis, particularly in understanding responses, health, vaccination, global polarization, and governance within the context of politics. In conclusion, our study not only provides valuable insights into public sentiment but also demonstrates the potential of AI in crisis management and decision-making during public health emergencies.

## Use of AI tools declaration

The authors declare no Artificial Intelligence (AI) tools have been used in the creation of this article.
